# PReS-FINAL-2098: Evaluation of the disease course of Italian children with juvenile idiopathic arthritis treated with etanercept: preliminary results in 313 patients

**DOI:** 10.1186/1546-0096-11-S2-P110

**Published:** 2013-12-05

**Authors:** S Verazza, A Consolaro, A Frisina, G Conti, D Rigante, C Robbiano, MG Alpigiani, A Ravelli

**Affiliations:** 1 Gaslini-Genova, Genoa, Italy; 2 Policlinico Messina, Messina, Italy; 3 Policlinico Gemelli, Rome, Italy

## Introduction

The advent of biologic medications has considerably increased the potential for treatment benefit in juvenile idiopathic arthritis (JIA), with clinical remission being now achievable in a substantial proportion of patients.

## Objectives

To evaluate the outcome of etanercept (ETN) therapy in Italian children with JIA.

## Methods

This is a multicenter, observational study that includes all children with JIA who were given ETN at Italian pediatric rheumatology centers after January 2000. Patients were classified in 2 groups: 1) children who were no longer taking ETN at study start; 2) patients who were still receiving ETN at study start. Patients in Group 1 underwent only retrospective assessments, whereas patients in Group 2 underwent both retrospective and cross-sectional assessments. The primary outcome of the study were reasons for ETN discontinuation in patients in Group 1, and achievement of the states of inactive disease (ID), minimal disease activity (MDA) and parent- and child-acceptable symptom state (PASS, CASS) in patients in Group 2. The above states were assessed through both formal definitions and JADAS cutoffs. The secondary outcome was the evaluation of frequency and characteristics of ETN-related side effects.

## Results

Twenty-five centers were asked to make a census of all patients followed at the center who met the inclusion criteria for Group 1 or Group 2. A total of 1230 patients were included in the census. Of these patients, 624 were still receiving ETN (Group 2), whereas 606 had discontinued ETN (Group 1). So far, the data of 313 patients (145 in Group 1 and 168 in Group 2) have been collected. Among the 145 patients in Group 1, reasons for ETN discontinuation included disease remission (41.4%), lack of efficacy (31%), and side effects (25.3%). The results of assessment of disease state through formal definitions in 106 children of the 168 children in Group 2 who had already undergone the cross-sectional evaluation were the following: ID 44.3%, MDA 76.4%, PASS 75.2%, CASS 68.9%. The percentages of patients who reached the same disease states assessed through JADAS cutoffs were: ID 41.5%, MDA 50.9%, PASS 61.3%, CASS 56.6%. Serious adverse events were seen in 8 of the 313 patients and included inflammatory bowel disease (3 pts), tuberculosis (1 pt), CMV hepatitis (1 pt), varicella complicated by bronchopneumonia (1 pt), bladder carcinoma (1 pt); 1 patient died of streptococcal sepsis.

## Conclusion

A substantial proportion of children currently receiving ETN were in the states of ID or MDA, or were satisfied with treatment outcome. The percentage of patients with ID in the cross-sectional cohort was comparable to the percentage of patients who were discontinued from ETN for disease remission in the retrospective cohort. Serious adverse events were uncommon.

## Disclosure of interest

None declared.

